# The Challenge and Opportunity to Diagnose Parkinson's Disease in Midlife

**DOI:** 10.3389/fneur.2019.01328

**Published:** 2019-12-17

**Authors:** Alexander Kilzheimer, Thomas Hentrich, Simone Burkhardt, Julia M. Schulze-Hentrich

**Affiliations:** Institute of Medical Genetics and Applied Genomics, University of Tübingen, Tübingen, Germany

**Keywords:** Parkinson's disease, prodromal, midlife, molecular prodrome, biomarker, diagnosis

## Abstract

Parkinson's disease (PD) is the most common neurodegenerative movement disorder that affects extensive regions of the nervous system. Its current clinical diagnosis is based on motor symptoms that appear late during disease progression when substantial proportions of the nigrostriatal dopaminergic neuron population are lost already. Although disturbances in sleep and other biofunctions often surface years prior to motor impairments and point to a long prodromal phase, these phenotypic signs in a person's midlife lack predictive power. They do, however, signal the unfolding of the disease and suggest molecular correlates that begin deviating early on. Revealing such trajectories, hence, promises not only a better understanding of prodromal PD but may also enable a much-needed earlier diagnosis. A nexus that may harbor such molecular trajectories is the epigenome as key etiological factors of PD—genetics, age, and environment—influence this substrate. An earlier diagnosis would also allow earlier interventions and lifestyle adjustments to improve brain function and reduce symptoms. In this review, we describe the challenges of diagnosing PD early on and highlight the opportunities that may arise from steering research efforts towards comprehensive interrogations of molecular layers during the long-time neglected midlife phase. In particular, we emphasize how existing cohorts of at-risk individuals, available animal models, and suitable markers may come together and aid in revealing molecular trajectories that offer diagnostic utility for PD in its prodromal stage.

## Introduction

Neurodegenerative disorders are a growing health threat in demographically aging societies. The complexity of many of these disorders is stereotypically exemplified in Parkinson's disease (PD), the second most common neurodegenerative disorder after Alzheimer's disease (1–4). Numbers of PD patients globally have more than doubled between 1990 and 2015 to an estimated 6.2 million and are predicted to affect more than 14 million individuals by 2040 (5–7).

The pathological hallmark of PD are misfolded alpha-synuclein protein (aSyn) structures accumulating in cellular inclusions known as Lewy bodies (8). Identifying neurons with aSyn inclusions in post-mortem pathological examinations of the brain is considered the gold standard for diagnosing PD (7, 9).

While about 10% of PD cases are familial and can be traced to specific genetic defects either in the *SNCA* locus that encodes aSyn (10) or in few other genes such as *LRRK2* (11), the vast majority derives from an unclear etiology. For these idiopathic cases, an unresolved interplay of genetic predisposition, age-related processes, and environmental as well as behavioral factors is assumed to be responsible for disease manifestation (12, 13). While several environmental and lifestyle factors, including exposure to pesticides, intake of beta blockers, or head trauma seemingly increase risk for disease onset and progression (14), age is the single most influential risk factor for PD (15, 16).

The prevalence rate of PD increases with age and rises from about 1% in people aged 60+ to 3.5% in individuals of 85–89 years of age (16–18). Prior to clinical diagnosis, typically at the age of 55–65, most idiopathic patients experience years or sometimes decades of unspecific symptomology, indicating the existence of a long prodromal phase. While these prodromal symptoms lack diagnostic specificity (19), they signal an early unfolding of pathology and, hence, are likely accompanied by molecular correlates that deviate from the healthy norm. Revealing such deviating molecular trajectories would greatly complement phenotypic markers and help to arrive at an earlier diagnosis than with today's criteria based primarily on motor symptoms. Earlier diagnosed patients would then benefit from engaging in neuroprotective lifestyle adjustments as well as optimized pharmacological treatments.

In this review, we stress the need and opportunity for diagnosing PD in its prodromal phase and summarize existing findings in order to identify neglected aspects and steer future research that might allow advancing from late- to early-stage PD diagnostics.

## Clinical Parkinson's Disease

PD is a progressive neurodegenerative disease that manifests in selective loss of dopaminergic neurons starting in early disease stages (20) and affects an array of motor and non-motor functions (21, 22). This loss is most prominent in the substantia nigra pars compacta, from which important afferent projections for the basal ganglia originate (23). Within the substantia nigra, the caudal and ventrolateral tier that project into the dorsal putamen of the striatum are usually affected most (24). The successive dopamine deficiency particular in this area is most likely causal for the development of characteristic motor features (25). However, neuronal loss in PD is not restricted to the basal ganglia, but also takes place in other brain regions including the hypothalamus, amygdala, locus coeruleus, raphe nucleus, nucleus basalis of Meynert, pedunculopontine nucleus, and dorsal motor nucleus of the vagus nerve (26). In addition, molecular hallmarks of PD can be found in non-brain compartments of the nervous system, such as the spinal cord, vagus nerve, sympathetic ganglia, cardiac plexus, enteric nervous system, adrenal medulla, and cutaneous nerves (27–30). Aside from dopamine, non-dopaminergic pathways seem to be affected by PD, too (31).

Similarly, clinical symptoms of PD are as diverse as the affected tissues, with motor impairments being the most prominent and characterizing deficits. Specifically, resting tremor, bradykinesia, rigidity, and postural instability are regarded as indicative features of PD (32, 33), with bradykinesia having the strongest correlation with the degree of dopamine deficiency (34). Clinically, PD is diagnosed primarily on motor symptoms (35) that surface in advanced disease stages when substantial proportions of the nigrostriatal dopaminergic neurons are lost (36–38).

## Prodromal Parkinson's Disease

**Figure 1:**
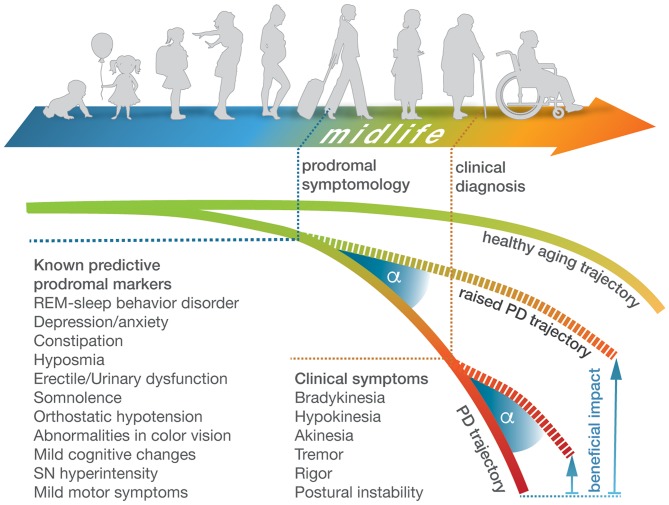
Prodromal and clinical symptomology of Parkinson's disease over the course of life. Schematic representation of the course of life from early childhood to old age and associated brain health trajectories that deviate in PD patients from the healthy norm (upper and lower curves). Current clinical diagnosis of PD is based on hallmark motor phenotypes that occur in advanced disease stages. They are preceded by prodromal symptomology that surfaces years to decades earlier during midlife but is less specific and varies considerably with respect to timing and features between individuals (44). Tracing deviating molecular trajectories from clinical stages into midlife would enable a prodromal diagnosis of PD, thereby widening the beneficial impact (shown as angle under raised brain health trajectories) of neuroprotective lifestyle adjustments or treatment options.

In addition to motor impairments, symptoms of PD typically involve a plethora of non-motor features that range from sensory disturbances, to neuropsychiatric and cognitive symptoms, to sleep disorders, and to autonomic dysfunction (39–41). Olfactory impairment/hyposmia, rapid-eye movement sleep behavior disorder (RBD), constipation, impairments in memory retrieval and decision making as well as depression (42–45) often have a greater impact on the patient's quality of life than motor deficits (46). A number of these symptoms precede motor deficits by years or even decades (Figure 1) (23, 47–49), but due to the lack of specificity, diagnostic possibilities based on these markers are still limited (19). However, for research purposes, many markers have been summarized in the Movement Disorder Society (MDS) research criteria for prodromal Parkinson's disease (50), in order to estimate the probability of prodromal PD. Among them are risk markers such as sex, caffeine consumption, smoking behavior, genetic mutations, and a positive family history, as well as prodromal markers, including RBD, olfactory loss, depression, and erectile dysfunction.

## The Need in Research to Focus on Midlife in Parkinson's Disease

The prodromal stage of PD coincides with midlife, approximately between 40 and 60 years of age (51). Midlife is a highly relevant period for biopsychosocial development with high interindividual heterogeneity and long-term impact for health in later life phases (51). In particular, the health status of the nervous system is influenced by lifestyle choices during midlife. Extensive stress during midlife, for example, correlates with self-care disability in older age (52). In contrast, individuals who exercise regularly in midlife have higher speed of cognitive processing, better memory and executive functions, and reduced risk of dementia later in life (53, 54). In addition, supportive social relationships and positive control beliefs in midlife support functional health and cognitive skills and curtail age-related health decline (55, 56). Regarding behavioral influences specific to PD, a physically and mentally active lifestyle in midlife is associated with a lower disease incidence and capable to reduce risk for developing PD by up to 40% (57–60). Hence, diagnosing PD in midlife would allow taking advantage of the neuroprotective potential of beneficial behavioral and environmental factors, most importantly by promoting physical activity. Pharmacological interventions might profit from a wider therapeutic time window that may lead to increased efficacy, too (61).

**Figure 2:**
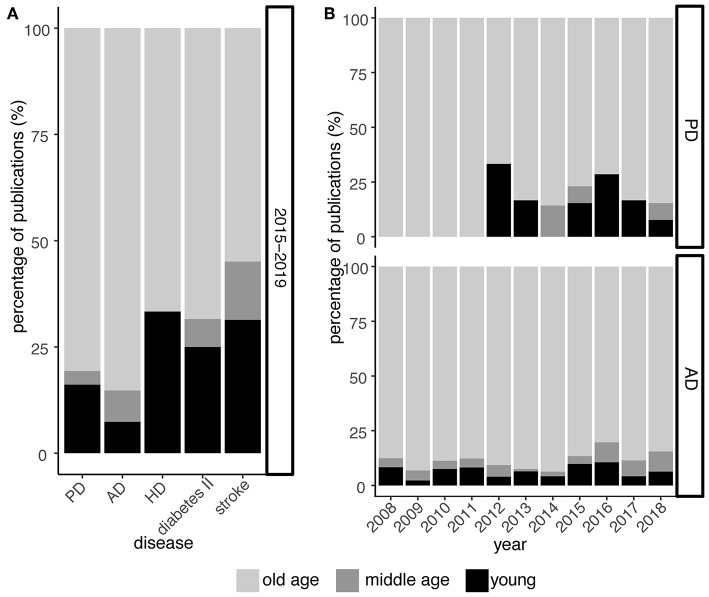
Midlife is still largely neglected in PD research. **(A)** Relative publication shares over the past 5 years (2015–2019) examining PD, Alzheimer's disease (AD), Huntington's disease (HD), type II diabetes, and stroke with respect to young, middle, and old age. **(B)** As in **(A)** but focusing on PD and AD at greater temporal resolution for the past 10 years. While research on the role of midlife in context of AD is continuously increasing, this trend is less obvious and seemingly lagging behind for PD. Numbers are derived from PubMed searches, querying publication titles and abstracts. For diseases, the terms [parkinson*] for PD, [alzheimer*] for AD, [huntington*] for HD, [*maturity-onset diabetes, noninsulin-dependent diabetes mellitus, diabetes mellitus type II, type 2 diabetes mellitus*] for type II diabetes, and [*ischemic stroke, cerebrovascular accident, cerebral infarction*] for stroke were used. For the “young” phase, the terms [adolescen*, youth*, juven*, young adult*, early*life, early adulthood, childhood], for the “middle age” phase, the terms [mid*life, middle*age*, mid*adulthood, middle*adulthood] and for the “old age” phase, the terms [late life, old*age*, senescence, elder*, senior*, geriatr*, *aged*] were used, respectively.

The role of midlife in PD, however, has largely been neglected by research so far (Figure 2A). Despite publication shares on PD and related neurodegenerative disorders like Alzheimer's and Huntington's disease indicate a focus on old age, the Alzheimer's field already shows rising awareness for midlife as increasing publication shares suggest (Figure 2B). In PD, this paradigm shift is not obvious yet but essential to embrace in order to derive a comprehensive understanding of the prodrome in PD.

## Molecular Markers of Parkinson's Disease

Research has explored a number of potential molecular markers for PD that center on measures that directly or indirectly assess the degree of mitochondrial dysfunction, oxidative stress, Lewy bodies formation, neuroinflammation, and other core pathological characteristics of PD in readily accessible biofluids or biopsies (62). Their utility in diagnosing or prognosing the disease early on, however, has remained limited (63, 64). Even for aSyn and its pivotal role in the etiology of familial and sporadic PD, its protein level in cerebrospinal fluid shows no correlation with progression of clinical motor symptoms (65). Similarily in saliva, in which aSyn can be detected, its concentration does not differ between PD patients and healthy controls (66). While even Lewy bodies have been found in salivary glands of PD patients (30), they too lack sensitivity and specificity (67). And neither in blood, for which aSyn (68), EGF (69), and several cytocines (70) have been considered, tests are conclusive. Interestingly, a growing amount of data suggests that aSyn may also accumulate in skin nerves of patients with PD and related diseases such as dementia with Lewy bodies and idiopathic RBD (71–75). These observations are currently being followed up with respect to their utility as potential biomarkers.

Despite these research efforts of the last decades that identified a wealth of candidate molecular readouts, there is no single reliable biomarker available yet that provides clinical utility for diagnosing or prognosing PD (76). This issue may largely result from the relatively low prevalence of PD on which sensitivity, specificity, and the predictive values of these measures are dependent (77) and has been discussed before (78–80).

## The Promising Field of Epigenetic Biomarkers

While suggestions exist to combine multiple markers in order to improve their diagnostical accuracy and to better capture the complexity of the disease (62), other approaches seek to increase the predictive power by focusing on molecular substrates on which several etiological or pathological aspects converge. With respect to the latter line of research, the epigenome has gained increasing research attention as major etiological contributors of the disease—genetics, age, and environment—are known to influence epigenetic modifications such as DNA methylation (81). The metastable nature of some epigenetic marks is both dynamic and yet sufficiently stable to capture and record environmental influences and behavioral stimuli that allow tracing disease pathology (82, 83).

Also with respect to the passage of time, the epigenome undergoes distinctive changes (84) and allows intriguingly accurate readouts of biological age through so-called epigenetic clocks that are based on DNA methylation at particular CpG sites in the genome (85–87). The accuracy and robustness of some of these clocks with respect to sex and tissue type (87, 88) led to their consideration as diagnostic readout for biological age in the context of PD, given that the disease has been suggested to represent a form of accelerated aging (89). Indeed, the epigenetic clock in blood of clinical PD cases indicates advanced age (90), although the deviation between chronological and biological age compared to effect sizes for related neurodegenerative diseases is much smaller (91). Nevertheless, genes near such clock sites are also often linked to PD and other neurodegenerative diseases (92).

These and other findings support the idea that epigenetic regulation is highly relevant in the context of complex neurodegenerative disease, especially in PD (93, 94), and that the epigenome represents a nexus which allows identifying epigenetic traces of early molecular deviations long before protein aggregation, widespread neuronal loss, and other characterizing features of advanced disease stages dominate molecular disturbances (95).

Nevertheless, while the epigenome may yield diagnostic markers for PD, it also harbors challenges and limitations. A critical challenge is that the magnitude of epigenetic changes in patients has to exceed the variation within populations and cell composition of the investigated tissue. Standardization has to be improved in order to reduce technical noise and evaluate the most suitable of the available interrogation technologies.

## Longitudinal Studies of at-risk Individuals

In order to reveal epigenetic traces as a proxy for deviating molecular trajectories during prodromal PD stages, comprehensive interrogations of epigenomic layers are required. While there is rich molecular data available covering the transcriptome, the epigenome, and even the metabolome for advanced disease stages (96–98), similar datasets for preclinical and prodromal stages of PD are still limited. Closing this gap, hence, requires a research shift from molecular interrogations in clinically diagnosed PD patients in advanced disease stages to early disease stages and, most importantly, at-risk individuals with prodromal symptomology in midlife. To address this need, some cohorts have already been established over the past few years (7). Among others, there are the:

- Parkinson's Progression Markers Initiative/PPMI (99): The study consists of 400 newly diagnosed PD patients and 200 healthy subjects that are being assessed for clinical, imaging, and molecular markers in urine, DNA, serum, plasma, RNA, whole blood, and cerebrospinal fluid.
- Prospective Validation of Risk Factors for the Development of Parkinsonian Syndromes/PRIPS (100): 1847 at baseline PD-free participants stratified by age, sex, family history, olfaction, motor functions, and substantia nigra echogenicity.
- Tübingen Evaluation of Risk Factors for Early Detection of Neurodegeneration/TREND (101): At baseline, this study enlists about 1,200 healthy individuals with no or at least one of three prodromal PD symptoms from depression, rapid eye movement behavior disorder, and hyposmia.
- The Parkinson's Associated Risk Study/PARS (102): A cohort of 303 hyp- and normosmic subjects without baseline diagnosis of PD.

These cohorts provide rich prior-to-diagnosis data from at-risk individuals that may develop PD during the observation period or that have just been diagnosed. Studies based on these cohorts focus on developments of prodromal symptomology and epidemiologic characteristics, but many of them also collect biological samples for molecular interrogations and profiling. Intensifying these efforts towards a better understanding of the molecular prodrome is crucial in order to tell apart typical age-related adaptations from disease-relevant molecular changes that might point at distinctive markers.

## Modeling Parkinson's Disease in Animals

Any studies on these promising cohorts, however, will still remain restricted to measures available through biofluids or biopsies. Inferring disease progression indirectly from these measures and not being able to directly profile disease unfolding on molecular level in the brain remains a fundamental limitation in neurodegenerative disease research. That is why animal models are inevitable as they uniquely enable studying any disease-relevant tissue directly and longitudinally over disease progression. In addition, they allow assessing environmental and behavioral influences on etiologically complex diseases such as PD through standardization and fine-grained monitoring of living conditions. However, this approach comes with the complex question of what exactly is being modeled. While it is possible to induce aSyn overexpression, Lewy bodies-like structures, or motor-deficient phenotypes, they only partially recapitulate the underlying molecular mechanism, histopathology, and phenotype of the disease. As with any model, of course, this reduction leads to comparability problems between the genuine disease and its models (103).

Despite these limitations, there are commonly accepted animal models of PD for a wide range of organisms along the evolutionary tree from worms and flies to non-human primates (104–106). Among them, rodent models lend themselves to research due to their relatively short lifespan but still sufficiently high organismal complexity.

## Toxin-Induced Animal Models of Parkinson's Disease

For PD rodent models, a common approach to induce pathology is to specifically target dopaminergic neurons with exogenous neurological toxins. Compounds such as 6-hydroxydopamine (6-OHDA) and 1-methyl-4-phenyl-1,2,3,6-tetrahydropyridine (MPTP) administered over days to weeks lead to loss of dopaminergic neurons and developments of PD-like phenotypes (107). The severity and extent of symptoms depend on dosage, mode of administration, and choice of rodent strain, thus allowing a certain combinatorial flexibility to mimic different and rather advanced stages of the disease (108, 109). As toxin-induced models typically lack aSyn inclusions (110) and starkly differ in their rapid loss of dopaminergic neurons from the much slower neuronal demise in patients, their utility for understanding prodromal PD remains limited (111, 112).

## Transgenic Models of Parkinson's Disease

Complementing toxin-induced models, transgenic animals that carry genetic defects linked to familial forms of PD represent an alternative approach to study the disease. Besides models with mutations in key loci including *LRRK2, PINK1, PRKN*, or *DJ-1* (113), those with defects in *SNCA* belong to the most commonly used ones. The human wildtype *SNCA*-overexpressing rat model (114), for example, represents a system that displays aSyn inclusions as well as a loss of dopaminergic neurons in an age-dependent manner accompanied by phenotypic impairments in sense of smell, increased anxiety-like behavior (115), and motor deficits later on (114). Genome-wide molecular investigations in a mouse model carrying the same transgenic construct suggest distinctive disturbance modes of deviating gene expression trajectories that underlie the earliest stages of *SNCA*-related pathology (116) and their modulation through environmental conditions (117, 118). By capturing early characteristics of pathology, these rodent models are also suited to investigate epigenetic mechanisms that presumably play a role in integrating and transducing external stimuli into the genomic regulatory program and form the basis of disease-modulating gene-environment interactions.

Lastly, combinations of toxin-induced and transgenic models exist, too. Examples are the LPS-treated Parkin-deficient mouse model (119) or the rotenone-treated LRRK2^R1441G^ knock-in mice line (120), which seek to unite the advantages of both modeling approaches and are geared to reflect larger parts of PD pathogenesis.

## Discussion

Taken together, the current clinical diagnosis of PD is primarily based on motor symptoms that occur at advanced disease stages when neuroprotective and risk-mitigating lifestyle adjustments are capped because of wide-spread neuronal loss (36–38). Distinctive phenotypic changes, however, already surface up to 20 years earlier and suggest unfolding of early pathogenic processes in midlife. Yet, these phenotypic characteristics alone lack discriminative power to diagnose PD in its so-called prodromal stage (19). To overcome this hurdle and substantially advance PD diagnostics, the underlying molecular prodrome in midlife requires greater research attention. Given that sequencing technology allows genome-wide molecular interrogations of unprecedented depth and breadth down to the level of single cells at ever-decreasing cost, research needs to fully embrace the paradigm shift and look prospectively at this alleged old-age disease. Cohorts enriched for at-risk individuals in tandem with animal models that facilitate paired measurements of brain and peripheral pathology in controlled environmental conditions have great potential to tackle the complexity of the disease from a new angle and to identify novel biomarkers. With respect to the latter, the epigenome should receive particular attention as individual aetiological contributors are already known to influence epigenetic marks. If this holds true also in context of early disease stages, distinctive epigenetic signatures might be the sought-after molecular complement to the phenotypic prodromal symptomology. Together, they may translate into a pheno-molecular tool of clinical utility that not only allows diagnosing prodromal PD but also offers a monitorable readout for disease progression and assessment of pharmacological treatments.

## Author Contributions

AK drafted the manuscript. SB did the PubMed data analysis. AK, TH, and JS-H wrote, reviewed, and edited the manuscript. JS-H conceived and coordinated the work. All authors have approved the final version.

### Conflict of Interest

The authors declare that the research was conducted in the absence of any commercial or financial relationships that could be construed as a potential conflict of interest.

## References

[1] Heinzel S Roeben B Ben-Shlomo Y Lerche S Alves G Barone P. Prodromal markers in Parkinson's disease: limitations in longitudinal studies and lessons learned. Front Aging Neurosci. (2016) 8:147. 10.3389/fnagi.2016.00147 27445791 PMC4916171

[2] Dorsey ER Constantinescu R Thompson JP Biglan KM Holloway RG Kieburtz K. Projected number of people with Parkinson disease in the most populous nations, 2005 through 2030. Neurology. (2007) 68:384–6. 10.1212/01.wnl.0000247740.47667.03 17082464

[3] Chin-Chan M Navarro-Yepes J Quintanilla-Vega B. Environmental pollutants as risk factors for neurodegenerative disorders: Alzheimer and Parkinson diseases. Front Cell Neurosci. (2015) 9:124. 10.3389/fncel.2015.00124 25914621 PMC4392704

[4] Obeso JA Stamelou M Goetz CG Poewe W Lang AE Weintraub D. Past, present, and future of Parkinson's disease: a special essay on the 200th Anniversary of the Shaking Palsy. Mov Disord. (2017) 32:1264–310. 10.1002/mds.27115 28887905 PMC5685546

[5] Bach J-P Ziegler U Deuschl G Dodel R Doblhammer-Reiter G. Projected numbers of people with movement disorders in the years 2030 and 2050. Mov Disord. (2011) 26:2286–90. 10.1002/mds.23878 22021158

[6] Dorsey ER Bloem BR. The Parkinson pandemic—a call to action. JAMA Neurol. (2018) 75:9–10. 10.1001/jamaneurol.2017.3299 29131880

[7] Rees RN Acharya AP Schrag A Noyce AJ An early diagnosis is not the same as a timely diagnosis of Parkinson's disease. F1000Res. (2018) 7:1106 10.12688/f1000research.14528.1 PMC6053699 30079229

[8] Goedert M Spillantini MG Del Tredici K Braak H. 100 years of Lewy pathology. Nat Rev Neurol. (2013) 9:13–24. 10.1038/nrneurol.2012.242 23183883

[9] Rizzo G Copetti M Arcuti S Martino D Fontana A Logroscino G. Accuracy of clinical diagnosis of Parkinson disease. Neurology. (2016) 86:566–76. 10.1212/WNL.0000000000002350 26764028

[10] Klein C Westenberger A. Genetics of Parkinson's disease. Cold Spring Harb Perspect Med. (2012) 2:a008888. 10.1101/cshperspect.a008888 22315721 PMC3253033

[11] Rui Q Ni H Li D Gao R Chen G. The role of LRRK2 in neurodegeneration of Parkinson disease. Curr Neuropharmacol. (2018) 16:1348–57. 10.2174/1570159X16666180222165418 29473513 PMC6251048

[12] Kieburtz K Wunderle KB. Parkinson's disease: evidence for environmental risk factors. Mov Disord. (2013) 28:8–13. 10.1002/mds.25150 23097348

[13] Racette BA Willis AW. Time to change the blind men and the elephant approach to Parkinson disease? Neurology. (2015) 85:190–6. 10.1212/WNL.0000000000001739 26070339 PMC4515036

[14] Bellou V Belbasis L Tzoulaki I Evangelou E Ioannidis JPA. Environmental risk factors and Parkinson's disease: an umbrella review of meta-analyses. Parkinsonism Relat Disord. (2016) 23:1–9. 10.1016/j.parkreldis.2015.12.008 26739246

[15] Emamzadeh FN Surguchov A. Parkinson's disease: biomarkers, treatment, and risk factors. Front Neurosci. (2018) 12:612. 10.3389/fnins.2018.00612 30214392 PMC6125353

[16] de Lau LM Breteler MM. Epidemiology of Parkinson's disease. Lancet Neurol. (2006) 5:525–35. 10.1016/S1474-4422(06)70471-9 16713924

[17] de Rijk MC Tzourio C Breteler MM Dartigues JF Amaducci L Lopez-Pousa S. Prevalence of parkinsonism and Parkinson's disease in Europe: the EUROPARKINSON Collaborative Study. European Community Concerted Action on the Epidemiology of Parkinson's disease. J Neurol Neurosurg Psychiatry. (1997) 62:10–5. 10.1136/jnnp.62.1.10 9010393 PMC486688

[18] Twelves D Perkins KSM Counsell C. Systematic review of incidence studies of Parkinson's disease. Mov Disord. (2003) 18:19–31. 10.1002/mds.10305 12518297

[19] Berg D Postuma RB. From prodromal to overt Parkinson's disease: towards a new definition in the year 2040. J Parkinsons Dis. (2018) 8:S19–23. 10.3233/JPD-181457 30584153 PMC6311373

[20] Kordower JH Olanow CW Dodiya HB Chu Y Beach TG Adler CH. Disease duration and the integrity of the nigrostriatal system in Parkinson's disease. Brain. (2013) 136:2419–31. 10.1093/brain/awt192 23884810 PMC3722357

[21] Alexander GE DeLong MR Strick PL. Parallel organization of functionally segregated circuits linking basal ganglia and cortex. Annu Rev Neurosci. (1986) 9:357–81. 10.1146/annurev.ne.09.030186.002041 3085570

[22] Lang AE Lozano AM Parkinson's disease. N Engl J Med. (1998) 339:1044–53. 10.1056/NEJM199810083391506 9761807

[23] Kalia LV Lang AE. Parkinson's disease. Lancet. (2015) 386:896–912. 10.1016/S0140-6736(14)61393-3 25904081

[24] Jellinger KA. Neuropathology of sporadic Parkinson's disease: evaluation and changes of concepts. Mov Disord. (2012) 27:8–30. 10.1002/mds.23795 22081500

[25] Dickson DW Braak H Duda JE Duyckaerts C Gasser T Halliday GM. Neuropathological assessment of Parkinson's disease: refining the diagnostic criteria. Lancet Neurol. (2009) 8:1150–7. 10.1016/S1474-4422(09)70238-8 19909913

[26] Dickson DW. Parkinson's disease and parkinsonism: neuropathology. Cold Spring Harb Perspect Med. (2012) 2:a009258. 10.1101/cshperspect.a009258 22908195 PMC3405828

[27] Beach TG Adler CH Sue LI Vedders L Lue L White CL III. Multi-organ distribution of phosphorylated alpha-synuclein histopathology in subjects with Lewy body disorders. Acta Neuropathol. (2010) 119:689–702. 10.1007/s00401-010-0664-3 20306269 PMC2866090

[28] Fumimura Y Ikemura M Saito Y Sengoku R Kanemaru K Sawabe M. Analysis of the adrenal gland is useful for evaluating pathology of the peripheral autonomic nervous system in lewy body disease. J Neuropathol Exp Neurol. (2007) 66:354–62. 10.1097/nen.0b013e3180517454 17483692

[29] Iwanaga K Wakabayashi K Yoshimoto M Tomita I Satoh H Takashima H. Lewy body-type degeneration in cardiac plexus in Parkinson's and incidental Lewy body diseases. Neurology. (1999) 52:1269–71. 10.1212/WNL.52.6.1269 10214756

[30] Del Tredici K Hawkes CH Ghebremedhin E Braak H. Lewy pathology in the submandibular gland of individuals with incidental Lewy body disease and sporadic Parkinson's disease. Acta Neuropathol. (2010) 119:703–13. 10.1007/s00401-010-0665-2 20229352

[31] van der Heeden JF Marinus J Martinez-Martin P van Hilten JJ. Importance of nondopaminergic features in evaluating disease severity of Parkinson disease. Neurology. (2014) 82:412–8. 10.1212/WNL.0000000000000087 24391163

[32] Jankovic J. Parkinson's disease: clinical features and diagnosis. J Neurol Neurosurg Psychiatry. (2008) 79:368–76. 10.1136/jnnp.2007.131045 18344392

[33] Chaudhuri KR Healy DG Schapira AH National Institute for Clinical Excellence. Non-motor symptoms of Parkinson's disease: diagnosis and management. Lancet Neurol. (2006) 5:235–45. 10.1016/S1474-4422(06)70373-8 16488379

[34] Vingerhoets FJ Schulzer M Calne DB Snow BJ. Which clinical sign of Parkinson's disease best reflects the nigrostriatal lesion? Ann Neurol. (1997) 41:58–64. 10.1002/ana.410410111 9005866

[35] Zhang T-M Yu S-Y Guo P Du Y Hu Y Piao Y-S. Nonmotor symptoms in patients with Parkinson disease: a cross-sectional observational study. Medicine. (2016) 95:e5400. 10.1097/MD.0000000000005400 27977578 PMC5268024

[36] Bernheimer H Birkmayer W Hornykiewicz O Jellinger K Seitelberger F. Brain dopamine and the syndromes of Parkinson and Huntington. Cli Morphol Neurochem Correlations J Neurol Sci. (1973) 20:415–55. 10.1016/0022-510X(73)90175-5 4272516

[37] Marek K Innis R van Dyck C Fussell B Early M Eberly S. [123I]beta-CIT SPECT imaging assessment of the rate of Parkinson's disease progression. Neurology. (2001) 57:2089–94. 10.1212/WNL.57.11.2089 11739831

[38] Braak H Ghebremedhin E Rüb U Bratzke H Del Tredici K. Stages in the development of Parkinson's disease-related pathology. Cell Tissue Res. (2004) 318:121–34. 10.1007/s00441-004-0956-9 15338272

[39] Rodríguez-Violante M Zerón-Martínez R Cervantes-Arriaga A Corona T. Who can diagnose Parkinson's disease first? Role of pre-motor symptoms. Arch Med Res. (2017) 48:221–7. 10.1016/j.arcmed.2017.08.005 28882322

[40] Schrag A Horsfall L Walters K Noyce A Petersen I. Prediagnostic presentations of Parkinson's disease in primary care: a case-control study. Lancet Neurol. (2015) 14:57–64. 10.1016/S1474-4422(14)70287-X 25435387

[41] Stern MB Siderowf A. Parkinson's at risk syndrome: can Parkinson's disease be predicted? Mov Disord. (2010) 25(Suppl 1):S89–93. 10.1002/mds.22719 20187248

[42] Chen H Zhao EJ Zhang W Lu Y Liu R Huang X. Meta-analyses on prevalence of selected Parkinson's nonmotor symptoms before and after diagnosis. Transl Neurodegener. (2015) 4:1. 10.1186/2047-9158-4-1 25671103 PMC4322463

[43] Pont-Sunyer C Hotter A Gaig C Seppi K Compta Y Katzenschlager R. The onset of nonmotor symptoms in Parkinson's disease (The ONSET PDStudy). Mov Disord. (2015) 30:229–37. 10.1002/mds.26077 25449044

[44] Postuma RB Berg D. Prodromal Parkinson's disease: the decade past, the decade to come. Mov Disord. (2019) 34:665–75. 10.1002/mds.27670 30919499

[45] Siderowf A Lang AE. Premotor Parkinson's disease: concepts and definitions. Mov Disord. (2012) 27:608–16. 10.1002/mds.24954 22508279 PMC3335740

[46] Sjödahl Hammarlund C Hagell P Nilsson MH. Motor and non-motor predictors of illness-related distress in Parkinson's disease. Parkinsonism Relat Disord. (2012) 18:299–302. 10.1016/j.parkreldis.2011.10.015 22100143

[47] Castillo-Barnes D Ramírez J Segovia F Martínez-Murcia FJ Salas-Gonzalez D Górriz JM. Robust ensemble classification methodology for I123-ioflupane SPECT images and multiple heterogeneous biomarkers in the diagnosis of Parkinson's disease. Front Neuroinform. (2018) 12:53. 10.3389/fninf.2018.00053 30154711 PMC6102321

[48] Pellicano C Benincasa D Pisani V Buttarelli FR Giovannelli M Pontieri FE. Prodromal non-motor symptoms of Parkinson's disease. Neuropsychiatr Dis Treat. (2007) 3:145–52. 10.2147/nedt.2007.3.1.145 19300544 PMC2654529

[49] Sveinbjornsdottir S. The clinical symptoms of Parkinson's disease. J Neurochem. (2016) 139:318–24. 10.1111/jnc.13691 27401947

[50] Berg D Postuma RB Adler CH Bloem BR Chan P Dubois B. MDS research criteria for prodromal Parkinson's disease. Mov Disord. (2015) 30:1600–11. 10.1002/mds.26431 26474317

[51] Lachman ME Teshale S Agrigoroaei S. Midlife as a pivotal period in the life course. Int J Behav Dev. (2015) 39:20–31. 10.1177/0165025414533223 25580043 PMC4286887

[52] Kulmala J von Bonsdorff MB Stenholm S Törmäkangas T von Bonsdorff ME Nygård C-H. Perceived stress symptoms in midlife predict disability in old age: a 28-year prospective cohort study. J Gerontol Ser A. (2013) 68:984–91. 10.1093/gerona/gls339 23371968

[53] Chang M Jonsson PV Snaedal J Bjornsson S Saczynski JS Aspelund T The effect of midlife physical activity on cognitive function among older adults: AGES—Reykjavik Study. J Gerontol Ser A. (2010) 65A:1369–74. 10.1093/gerona/glq152 PMC2990266 20805238

[54] Defina LF Willis BL Radford NB Gao A Leonard D Haskell WL. The association between midlife cardiorespiratory fitness levels and later-life dementia: a cohort study. Ann Intern Med. (2013) 158:162–8. 10.7326/0003-4819-158-3-201302050-00005 23381040 PMC3926646

[55] Lachman ME Agrigoroaei S. Promoting functional health in midlife and old age: long-term protective effects of control beliefs, social support, and physical exercise. PLoS ONE. (2010) 5:e13297. 10.1371/journal.pone.0013297 20949016 PMC2952603

[56] Sabia S Singh-Manoux A Hagger-Johnson G Cambois E Brunner EJ Kivimaki M. Influence of individual and combined healthy behaviours on successful aging. C Can Med Assoc J. (2012) 184:1985–92. 10.1503/cmaj.121080 23091184 PMC3519184

[57] Goodwin VA Richards SH Taylor RS Taylor AH Campbell JL. The effectiveness of exercise interventions for people with Parkinson's disease: a systematic review and meta-analysis. Mov Disord. (2008) 23:631–40. 10.1002/mds.21922 18181210

[58] Hamer M Chida Y. Physical activity and risk of neurodegenerative disease: a systematic review of prospective evidence. Psychol Med. (2009) 39:3–11. 10.1017/S0033291708003681 18570697

[59] Thacker EL Chen H Patel AV McCullough ML Calle EE Thun MJ. Recreational physical activity and risk of Parkinson's disease. Mov Disord. (2008) 23:69–74. 10.1002/mds.21772 17960818 PMC2387117

[60] Xu Q Park Y Huang X Hollenbeck A Blair A Schatzkin A. Physical activities and future risk of Parkinson disease. Neurology. (2010) 75:341–8. 10.1212/WNL.0b013e3181ea1597 20660864 PMC2918886

[61] Zeuner KE Schäffer E Hopfner F Brüggemann N Berg D. Progress of pharmacological approaches in Parkinson's disease. Clin Pharmacol Ther. (2019) 105:1106–20. 10.1002/cpt.1374 30661251

[62] He R Yan X Guo J Xu Q Tang B Sun Q. Recent advances in biomarkers for Parkinson's disease. Front Aging Neurosci. (2018) 10:305. 10.3389/fnagi.2018.00305 30364199 PMC6193101

[63] Delenclos M Jones DR McLean PJ Uitti RJ. Biomarkers in Parkinson's disease: advances and strategies. Parkinsonism Relat Disord. (2016) 22:S106–10. 10.1016/j.parkreldis.2015.09.048 26439946 PMC5120398

[64] Miller DB O'Callaghan JP. Biomarkers of Parkinson's disease: present and future. Metabolism. (2015) 64:S40–6. 10.1016/j.metabol.2014.10.030 25510818 PMC4721253

[65] Stewart T Liu C Ginghina C Cain KC Auinger P Cholerton B. Cerebrospinal fluid α-synuclein predicts cognitive decline in parkinson disease progression in the DATATOP cohort. Am J Pathol. (2014) 184:966–75. 10.1016/j.ajpath.2013.12.007 24625392 PMC3969999

[66] Devic I Hwang H Edgar JS Izutsu K Presland R Pan C. Salivary α-synuclein and DJ-1: potential biomarkers for Parkinson's disease. Brain. (2011) 134:e178. 10.1093/brain/awr015 21349902 PMC3122368

[67] Folgoas E Lebouvier T Leclair-Visonneau L Cersosimo M-G Barthelaix A Derkinderen P. Diagnostic value of minor salivary glands biopsy for the detection of Lewy pathology. Neurosci Lett. (2013) 551:62–4. 10.1016/j.neulet.2013.07.016 23880024

[68] Ravina B Tanner C Dieuliis D Eberly S Flagg E Galpern WR. A longitudinal program for biomarker development in Parkinson's disease: a feasibility study. Mov Disord. (2009) 24:2081–90. 10.1002/mds.22690 19691116

[69] Lim NS Swanson CR Cherng H-R Unger TL Xie SX Weintraub D. Plasma EGF and cognitive decline in Parkinson's disease and Alzheimer's disease. Ann Clin Transl Neurol. (2016) 3:346–55. 10.1002/acn3.299 27231704 PMC4863747

[70] Choi C Jeong J-H Jang JS Choi K Lee J Kwon J. Multiplex analysis of cytokines in the serum and cerebrospinal fluid of patients with Alzheimer's disease by color-coded bead technology. J Clin Neurol. (2008) 4:84. 10.3988/jcn.2008.4.2.84 19513308 PMC2686871

[71] Donadio V Incensi A Rizzo G Capellari S Pantieri R Stanzani Maserati M. A new potential biomarker for dementia with Lewy bodies: Skin nerve α-synuclein deposits. Neurology. (2017) 89:318–26. 10.1212/WNL.0000000000004146 28667178

[72] Donadio V Incensi A El-Agnaf O Rizzo G Vaikath N Del Sorbo F. Skin α-synuclein deposits differ in clinical variants of synucleinopathy: an *in vivo* study. Sci Rep. (2018) 8:14246. 10.1038/s41598-018-32588-8 30250046 PMC6155202

[73] Donadio V Doppler K Incensi A Kuzkina A Janzen A Mayer G. Abnormal α-synuclein deposits in skin nerves: intra- and inter-laboratory reproducibility. Eur J Neurol. (2019) 26:1245–51. 10.1111/ene.13939 30770596

[74] Doppler K Jentschke H-M Schulmeyer L Vadasz D Janzen A Luster M. Dermal phospho-alpha-synuclein deposits confirm REM sleep behaviour disorder as prodromal Parkinson's disease. Acta Neuropathol. (2017) 133:535–45. 10.1007/s00401-017-1684-z 28180961 PMC5348554

[75] Gibbons CH Garcia J Wang N Shih LC Freeman R. The diagnostic discrimination of cutaneous α-synuclein deposition in Parkinson disease. Neurology. (2016) 87:505–12. 10.1212/WNL.0000000000002919 27385742 PMC4970658

[76] Rizek P Kumar N Jog MS. An update on the diagnosis and treatment of Parkinson disease. CMAJ. (2016) 188:1157–65. 10.1503/cmaj.151179 27221269 PMC5088077

[77] Brenner H Gefeller O. Variation of sensitivity, specificity, likelihood ratios and predictive values with disease prevalence. Stat Med. (1997) 16:981–91. 10.1002/(SICI)1097-0258(19970515)16:93.3.CO;2-E 9160493

[78] Montgomery EB. Predictors of Parkinson's disease–not quite sound. Mov Disord. (2013) 28:413–5. 10.1002/mds.25432 23483647

[79] Montgomery EB Koller WC LaMantia TJ Newman MC Swanson-Hyland E Kaszniak AW Early detection of probable idiopathic Parkinson's disease: I. Development of a diagnostic test battery. Mov Disord. (2000) 15:467–73. 10.1002/1531-8257(200005)15:3<467::AID-MDS1007>3.0.CO;2-# 10830410

[80] Montgomery EB Lyons K Koller WC. Early detection of probable idiopathic Parkinson's disease: II. A prospective application of a diagnostic test battery. Mov Disord. (2000) 15:474–8. 10.1002/1531-8257(200005)15:3<474::AID-MDS1008>3.0.CO;2-X 10830411

[81] van Dongen J Nivard MG Willemsen G Hottenga J-J Helmer Q Dolan CV. Genetic and environmental influences interact with age and sex in shaping the human methylome. Nat Commun. (2016) 7:11115. 10.1038/ncomms11115 27051996 PMC4820961

[82] García-Giménez JL Mena-Mollá S Beltrán-García J Sanchis-Gomar F. Challenges in the analysis of epigenetic biomarkers in clinical samples. Clin Chem Lab Med. (2017) 55:1474–7. 10.1515/cclm-2016-1162 28301317

[83] Moore DS. Behavioral epigenetics. Wiley Interdiscip Rev Syst Biol Med. (2017) 9:e1333. 10.1002/wsbm.1333 27906527

[84] Booth LN Brunet A. The aging epigenome. Mol Cell. (2016) 62:728–44. 10.1016/j.molcel.2016.05.013 27259204 PMC4917370

[85] Florath I Butterbach K Muller H Bewerunge-Hudler M Brenner H. Cross-sectional and longitudinal changes in DNA methylation with age: an epigenome-wide analysis revealing over 60 novel age-associated CpG sites. Hum Mol Genet. (2014) 23:1186–201. 10.1093/hmg/ddt531 24163245 PMC3919014

[86] Hannum G Guinney J Zhao L Zhang L Hughes G Sadda S. Genome-wide methylation profiles reveal quantitative views of human aging rates. Mol Cell. (2013) 49:359–67. 10.1016/j.molcel.2012.10.016 23177740 PMC3780611

[87] Horvath S. DNA methylation age of human tissues and cell types. Genome Biol. (2013) 14:R115. 10.1186/gb-2013-14-10-r115 24138928 PMC4015143

[88] Spiers H Hannon E Schalkwyk LC Smith R Wong CCY O'Donovan MC. Methylomic trajectories across human fetal brain development. Genome Res. (2015) 25:338–52. 10.1101/gr.180273.114 25650246 PMC4352878

[89] Collier TJ Kanaan NM Kordower JH. Aging and Parkinson's disease: different sides of the same coin? Mov Disord. (2017) 32:983–90. 10.1002/mds.27037 28520211 PMC5844262

[90] Horvath S Ritz BR. Increased epigenetic age and granulocyte counts in the blood of Parkinson's disease patients. Aging. (2015) 7:1130–42. 10.18632/aging.100859 26655927 PMC4712337

[91] Horvath S Langfelder P Kwak S Aaronson J Rosinski J Vogt TF. Huntington's disease accelerates epigenetic aging of human brain and disrupts DNA methylation levels. Aging. (2016) 8:1485–512. 10.18632/aging.101005 27479945 PMC4993344

[92] Lu AT Hannon E Levine ME Hao K Crimmins EM Lunnon K. Genetic variants near MLST8 and DHX57 affect the epigenetic age of the cerebellum. Nat Commun. (2016) 7:10561. 10.1038/ncomms10561 26830004 PMC4740877

[93] Feng Y Jankovic J Wu Y-C. Epigenetic mechanisms in Parkinson's disease. J Neurol Sci. (2015) 349:3–9. 10.1016/j.jns.2014.12.017 25553963

[94] Henderson-Smith A Fisch KM Hua J Liu G Ricciardelli E Jepsen K. (2019). DNA methylation changes associated with Parkinson's disease progression: outcomes from the first longitudinal genome-wide methylation analysis in blood. Epigenetics 14:365–382. 10.1080/15592294.2019.1588682 30871403 PMC6557551

[95] Jakubowski JL Labrie V. Epigenetic biomarkers for Parkinson's disease: from diagnostics to therapeutics. J Parkinsons Dis. (2017) 7:1–12. 10.3233/JPD-160914 27792016 PMC5302044

[96] Borrageiro G Haylett W Seedat S Kuivaniemi H Bardien S. A review of genome-wide transcriptomics studies in Parkinson's disease. Eur J Neurosci. (2018) 47:1–16. 10.1111/ejn.13760 29068110

[97] van Heesbeen HJ Smidt MP. Entanglement of genetics and epigenetics in Parkinson's disease. Front Neurosci. (2019) 13:277. 10.3389/fnins.2019.00277 30983962 PMC6449477

[98] Shao Y Le W. Recent advances and perspectives of metabolomics-based investigations in Parkinson's disease. Mol Neurodegener. (2019) 14:3. 10.1186/s13024-018-0304-2 30634989 PMC6330496

[99] Marek K Jennings D Lasch S Siderowf A Tanner C Simuni T The Parkinson Progression Marker Initiative (PPMI). Prog Neurobiol. (2011) 95:629–35. 10.1016/j.pneurobio.2011.09.005 21930184 PMC9014725

[100] Berg D Godau J Seppi K Behnke S Liepelt-Scarfone I Lerche S. The PRIPS study: screening battery for subjects at risk for Parkinson's disease. Eur J Neurol. (2013) 20:102–8. 10.1111/j.1468-1331.2012.03798.x 22852790

[101] Gaenslen A Wurster I Brockmann K Huber H Godau J Faust B. Prodromal features for Parkinson's disease - baseline data from the TREND study. Eur J Neurol. (2014) 21:766–72. 10.1111/ene.12382 24612314

[102] Jennings D Siderowf A Stern M Seibyl J Eberly S Oakes D. Imaging prodromal Parkinson disease: the Parkinson Associated Risk Syndrome Study. Neurology. (2014) 83:1739–46. 10.1212/WNL.0000000000000960 25298306 PMC4239830

[103] Montgomery EB Reproducibility in *Biomedical Research Epistemological and Statistical Problems*. London, UK: Academic Press (2019).

[104] Blesa J Trigo-Damas I del Rey NL-G Obeso JA. The use of nonhuman primate models to understand processes in Parkinson's disease. J Neural Transm. (2018) 125:325–35. 10.1007/s00702-017-1715-x 28357564

[105] Cooper JF Van Raamsdonk JM. Modeling Parkinson's disease in *C. elegans*. J Parkinsons Dis. (2018) 8:17–32. 10.3233/JPD-171258 29480229 PMC5836411

[106] Xiong Y Yu J. Modeling Parkinson's disease in drosophila: what have we learned for dominant traits? Front Neurol. (2018) 9:228. 10.3389/fneur.2018.00228 29686647 PMC5900015

[107] Terzioglu M Galter D. Parkinson's disease: genetic versus toxin-induced rodent models. FEBS J. (2008) 275:1384–91. 10.1111/j.1742-4658.2008.06302.x 18279376

[108] Meredith GE Rademacher DJ. MPTP mouse models of Parkinson's disease: an update. J Parkinsons Dis. (2011) 1:19–33. 10.3233/JPD-2011-11023 23275799 PMC3530193

[109] Tieu K. A guide to neurotoxic animal models of Parkinson's disease. Cold Spring Harb Perspect Med. (2011) 1:a009316. 10.1101/cshperspect.a009316 22229125 PMC3234449

[110] Athauda D Foltynie T. The ongoing pursuit of neuroprotective therapies in Parkinson disease. Nat Rev Neurol. (2015) 11:25–40. 10.1038/nrneurol.2014.226 25447485

[111] Mattace Raso G Avagliano C Calignano A. Response to comment by Juan Segura-Aguilar: new preclinical model are required to discover neuroprotective compound in Parkinson's disease. Pharmacol Res. (2017) 119:491–2. 10.1016/j.phrs.2016.11.026 27890814

[112] Segura-Aguilar J. New preclinical model are required to discover neuroprotective compound in Parkinson's disease. Pharmacol Res. (2017) 119:490. 10.1016/j.phrs.2016.11.034 27894922

[113] Dawson TM Golde TE Lagier-Tourenne C. Animal models of neurodegenerative diseases. Nat Neurosci. (2018) 21:1370–9. 10.1038/s41593-018-0236-8 30250265 PMC6615039

[114] Nuber S Harmuth F Kohl Z Adame A Trejo M Schönig K. A progressive dopaminergic phenotype associated with neurotoxic conversion of α-synuclein in BAC-transgenic rats. Brain. (2013) 136:412–32. 10.1093/brain/aws358 23413261 PMC3572936

[115] Kohl Z Ben Abdallah N Vogelgsang J Tischer L Deusser J Amato D. Severely impaired hippocampal neurogenesis associates with an early serotonergic deficit in a BAC α-synuclein transgenic rat model of Parkinson's disease. Neurobiol Dis. (2016) 85:206–17. 10.1016/j.nbd.2015.10.021 26523794 PMC4974940

[116] Hentrich T Wassouf Z Riess O Schulze-Hentrich JM. SNCA overexpression disturbs hippocampal gene expression trajectories in midlife. Aging. (2018) 10:4024–41. 10.18632/aging.101691 30543522 PMC6326667

[117] Wassouf Z Hentrich T Samer S Rotermund C Kahle PJ Ehrlich I. Environmental enrichment prevents transcriptional disturbances induced by alpha-synuclein overexpression. Front Cell Neurosci. (2018) 12:112. 10.3389/fncel.2018.00112 29755323 PMC5932345

[118] Wassouf Z Hentrich T Casadei N Jaumann M Knipper M Riess O. Distinct stress response and altered striatal transcriptome in alpha-synuclein overexpressing mice. Front Neurosci. (2019) 12:1033. 10.3389/fnins.2018.01033 30686992 PMC6336091

[119] Frank-Cannon TC Tran T Ruhn KA Martinez TN Hong J Marvin M. Parkin deficiency increases vulnerability to inflammation-related nigral degeneration. J Neurosci. (2008) 28:10825–34. 10.1523/JNEUROSCI.3001-08.2008 18945890 PMC2603252

[120] Liu H-F Ho PW-L Leung GC-T Lam CS-C Pang SY-Y Li L. Combined LRRK2 mutation, aging and chronic low dose oral rotenone as a model of Parkinson's disease. Sci Rep. (2017) 7:40887. 10.1038/srep40887 28098219 PMC5241661

